# The whole is greater than the sum of its parts: Integrating syndemics and intersectionality in tackling the HIV and mental health epidemics among Filipino gay and bisexual men

**DOI:** 10.1371/journal.pmen.0000252

**Published:** 2025-02-07

**Authors:** Aron Harold G. Pamoso, Mary Lou Rasmussen, I. Nyoman Sutarsa, Brett Scholz

**Affiliations:** 1 School of Medicine and Psychology, The Australian National University, Acton, Australian Capital Territory, Australia; 2 School of Sociology, The Australian National University, Acton, Australian Capital Territory, Australia; PLOS: Public Library of Science, UNITED KINGDOM OF GREAT BRITAIN AND NORTHERN IRELAND

## Abstract

Insufficient attention has been given to the social aspects of HIV and mental health in the Philippines despite their profound impact on Filipinos, particularly gay and bisexual men and other men who have sex with men. Past evidence shows that these health conditions are intertwined, amplified by the combined impact of power and oppression. Yet, scholarly works, programs, and interventions focusing on understanding the social, structural, and political aspects of HIV and mental health are still in the early stages in the Philippines, calling for urgency in tackling the ongoing epidemics these men face. Therefore, we have developed this essay with the following objectives: 1) to provide context for the overlapping of the two health epidemics faced by Filipino gay and bisexual men and men who have sex with men; 2) to illustrate how critical perspectives such as syndemics and intersectionality can deepen our understanding of these conditions; 3) to showcase strategies for integrating syndemics and intersectionality into research and practice. Our aim is to foster the discussion on the use of critical perspectives in addressing health conditions in the Philippines and to advocate for comprehensive, inclusive, and culturally informed research, programs, and interventions for these men in the broader Filipino community.

## Introduction

Sexual and gender minority populations continue to be disproportionately affected by HIV [1]. To address this ongoing health issue, the Joint United Nations Programme on HIV/AIDS UNAIDS [2] introduced a new goal, the 95-95-95 target. These targets aim for 95% of people living with HIV (PLHIV) to be aware of their HIV-positive status, 95% of those individuals to be on continuous antiretroviral therapy, and 95% of those on antiretroviral therapy to have their viral loads managed and suppressed [2]. This reflects the ongoing commitment of the global community to addressing AIDS and achieving the sustainable development goals of 2030, including “ending AIDS” as a public health issue [3].

Despite the global alliance to end AIDS by 2030, scholars predict that many countries will still be unable to achieve this goal due to limited resources on interventions in mitigating HIV [4]. Past evidence indicated that over the last 10 years, “progress in reducing new infections has been slow,” and resources to tackle HIV in low-income countries have only increased gradually [5]. In particular, the Philippines has experienced the highest growth of HIV in the Western Pacific Region, with a rise of 174% in incidence of HIV between 2010 and 2017 [6]. For context, in 2012, there were around nine people every day living with HIV [7], but from July to September of 2024, there were 50 people every day living with HIV [8]. This trend is a 411% increase in the daily incidence of HIV within 10 years. Experts estimated that PLHIV will grow from 158,400 in 2022 to 364,0000 in 2030 [9]. This burden of HIV is felt the most among the Filipino multiply marginalized communities. One key demographic–men and men who have sex with men (MSM)–was reported to have the highest number of people who are living with HIV [9]. Nonetheless, progress is visible in addressing the HIV epidemic in the Philippines through access to pre-exposure prophylaxis (PrEP), post-exposure prophylaxis (PEP), newer antiretroviral therapy (ART) agents, and reforms in legislation and policies aiming to address the ongoing HIV epidemic [7].

In the context of PrEP and PEP, the Philippines has implemented a PrEP demonstration project for men who have sex with men. Healthcare providers (e.g., medical doctors, counselors, nurses, volunteers) in HIV services demonstrated knowledge and provision of PrEP/PEP. However, they highlighted infrastructure-related issues that could hinder the implementation of PrEP/PEP [10]. Evidence indicated that Filipino MSM had a high level of knowledge and awareness about HIV and PrEP, particularly among individuals with higher educational attainment, higher income, and those who have undergone HIV testing [11]. Within the context of HIV testing, adherence to ART, and PrEP, the results of community-led online HIV self-testing initiatives suggest that they are safe and likely to increase HIV testing and linkage to care among Filipino MSM. However, evidence suggests that the “initiation of ART and PrEP were low” due to limited resources and cost, respectively [12].

Filipino MSM experience poor mental health outcomes compared to the general population, with a significantly higher risk of suicide [13]. Research suggests they are twice as likely to experience suicidal ideation than straight Filipino men [13]. Young adult LGBTQIA+ individuals also report higher levels of depression, anxiety and stress than heterosexual peers [14]. Alibudbud [15] found that risky sexual behaviors were positively associated with depression among Filipino MSM. Fear of stigma deters individuals with mental health issues from seeking help [16], and MSM—whether living with HIV or not—frequently avoid HIV care for the same reasons [17]. These findings highlight the interconnectedness of HIV and mental health challenges in this population, underscoring the structural elements that perpetuate health inequities. Despite the Philippines being considered relatively open towards LGBTQIA+ individuals [18], negative societal views persist, leading to perceived stress, internalized stigma, and increased mental health problems such as depression and suicidal thoughts [13,19]. Recent research highlighted how they actively navigate challenges by seeking support from peers, family, and social institutions, employing various coping strategies such as addressing issues related to their sexual identities and deepening their understanding of their identities [20].

When scholars view HIV and mental health as separate issues affecting the Filipino MSM, they may fail to address the full scope of their health needs. For instance, if we focus solely on addressing HIV through antiretroviral therapy (ART), we are primarily addressing the physical and clinical aspects of the health condition. However, as highlighted earlier in this paper, HIV often coexists with mental health conditions. Therefore, if we concentrate exclusively on HIV intervention, we may overlook mental health conditions and their symptoms, thereby missing the opportunity to provide comprehensive, holistic care for this population.

### Syndemics

Syndemics theory has emerged as a framework for understanding interactions between two or more health conditions and/or phenomena. These are often complex issues that are difficult to solve and highly influenced by socio-structural and political factors, such as disparities in healthcare access [21,22]. Syndemics could be understood as the “synergistic interaction of two or more coexistent diseases and resultant excess burden of the disease.” Scholars conceptualize syndemics theory into 1) the co-existence of two or more health conditions; 2) the synergy of the two or more health conditions using the biopsychosocial pathways [23]; and 3) how various social, structural, political, and psychological elements (e.g., racism, heterosexism, ableism, classism) sustains the interaction of the syndemic condition, i.e., the interaction of two or more health conditions [21,24]. Over two decades, the framework has been applied to tackle health inequities [e.g., 24], such as tuberculosis [e.g., 25] and COVID-19 [e.g., 26]. Through the lens of syndemics, health is understood as going beyond the biomedical aspects of disease. It considers the critical role of social and geopolitical elements that shape health outcomes [27,28]. We contend that this critical framework is particularly relevant for examining how the co-occurrence of HIV and mental health issues sustains poor health outcomes among Filipino MSM.

Although a syndemics approach provides an opportunity to explore the co-existence of HIV and poor mental health among Filipino MSM, it has limitations. Research on syndemics often fails to focus explicitly on social and structural contexts in which the health conditions interact [29]. Scholars critique its narrow biomedical focus, emphasizing clinical medicine while frequently overlooking how power dynamics and social inequities shape health outcomes [29]. Bulled, Singer, and Ostrach [30] argued that although syndemics theory acknowledges systemic oppression, little effort was made to describe how biological and social factors interact to worsen health outcomes [29,31].

When applying syndemics theory to Filipino MSM, researchers may focus on HIV or mental health individually without fully addressing how social and structural factors shape their interaction, thereby increasing vulnerability and exacerbating oppression. Additionally, the framework often neglects the overlap of stigmatized social identities and how systems of power fuel this stigma. For example, gay and bisexual men (GBM) living with HIV face multiple stigmas due to both their HIV-positive status and sexual identities [32].

Without emphasizing social and structural factors, the syndemics framework risks oversimplifying the complex interactions between health conditions and social identities. To address this limitation, scholars recommend complementing syndemics with other theories [31,33,34] or modifying them [35]. Quinn [29] suggested that incorporating intersectionality into syndemics research could attend “to issues of power and oppression, historical trauma, and structural violence that are not typically included in public health or syndemic research.” Filipino GBM living with HIV, for example, face stigma rooted in their HIV status and sexual identity. Consequently, these men experience poor mental health outcomes [15].

### Intersectionality

Intersectionality, as a critical theory, examines the inequities among multiply marginalized populations by exploring how intersecting systems of oppression and power shape their experiences [36–39]. It moves beyond merely categorizing stigmatized social identities, emphasizing how systemic forces contribute to these populations’ oppression. This complex conceptualization originated from the seminal work of Crenshaw [37,38]. Moreover, scholars [36] described intersectionality as a framework that illustrates the nuanced experiences of multiply marginalized populations by comparing it to a “matrix of domination characterized by intersecting oppressions” [p.3]. Building on this, researchers [40] argued that intersectionality should not focus on “single axis” perspectives of inequities but adopt a dynamic, “matrix” approach that prioritizes collective justice [p. 251].

Intersectionality comprises three elements: 1) the overlap of multiple stigmatized social identities; 2) the shaping of these identities by power imbalances and oppression; and 3) the interaction of these identities with socio-cultural contexts [41]. The theory provides activists and scholars with a powerful tool for examining how interconnected social identities influence the lives of historically marginalized populations [42]. We assert that intersectionality and other critical approaches to health challenge the conventional biomedical model of illness, which often categorizes individuals through risk theory. This model homogenizes experiences and decontextualizes their realities, placing people into rigid categories [43]. Intersectionality addresses these issues by amplifying their lived experiences [42] and focusing on the unique concerns of marginalized communities within their specific contexts [44].

Applying an intersectional lens to Filipino GBM living with HIV reveals that they face multiple stigmatizations: one rooted in their sexual identities and the other in their HIV status. These experiences are shaped by rigid sociocultural values, heavily influenced by Roman Catholicism, which permeates familial and social relationships [32]. Intersectionality contextualized their struggles and resilience, transforming their roles from victims of oppression to activists for social change within their communities, where they serve as volunteers and leaders promoting HIV awareness [32].

Utilizing intersectionality within the syndemics framework of HIV and mental health among Filipino MSM allows for an investigation into the deleterious synergy of HIV and mental health (i.e., syndemics). Simultaneously, it reveals how interlocking systems of power, such as heterosexism and religious values, fuel stigmatization and marginalization, thereby exacerbating the impact of HIV and mental health conditions.

Drawing upon syndemics and intersectionality, we have developed recommendations for how syndemics and intersectionality can inform stakeholders—scholars, healthcare workers, activists, and policymakers—about their research and praxis. Our goal is not to discredit existing models of care and intervention, including present theories within the existing HIV and mental health landscape in the Philippines, but rather to encourage stakeholders to embrace and integrate critical approaches to addressing the ongoing HIV and mental health issues in the Philippines.

These recommendations draw upon the diverse backgrounds of the authors. The first author is an openly gay Filipino scholar and HIV activist who researched stigma experiences among Filipino MSM living with HIV, Indigenous Filipino students, and the general Filipino population during the COVID-19 pandemic. The other authors have backgrounds in sociology, queer theory, lived experience leadership, public health, and primary healthcare settings. We acknowledge that all the authors are academics working in a relatively well-developed Western country, which we assume comes with privilege (although we note that parts of our identities exist in a matrix of privilege and relative marginalization). We embrace these characteristics and use them as a process to be reflexive in our experiences and privileges and how these experiences and privileges shape how we conceptualize and present this current paper.

### Recommendations

#### 1. Democratize expertise

To drive meaningful healthcare reforms in HIV and mental health and to integrate critical approaches to health (e.g., syndemics and intersectionality), it is essential to democratize expertise at both individual and systemic levels. We assert that making space for individuals with lived experience of health conditions to be part of organizational and systemic levels of the health sector—such as policymaking, decision-making, and co-designing programs and interventions—requires a bottom-up approach. By starting at the individual level, e.g., shared health decision-making, we can strengthen collaboration and foster their involvement in higher-level health system processes.

An example is how Filipinos, including GBM, are involved in health decision-making. In the current healthcare landscape, medical healthcare workers, such as doctors, are seen as the sole experts with the final say in their healthcare. However, recent evidence indicates that Filipinos want a voice in healthcare decisions [45]. We argue that the biomedical model is the primary healthcare framework practiced in the Philippines. As we explained in the earlier sections of this paper, the biomedical model tends to overlook the contexts of people’s experiences and treats everyone as the same [43]. We emphasize that this approach could result in a narrow focus on health and may lead to potential issues. For example, when healthcare workers are the sole authority in deciding the best course of action for individuals with HIV or mental health conditions, it may dismiss the experiences of the people living with or experiencing these health conditions. This could create a power imbalance between the patient and the healthcare worker, potentially leading to health inequities. As such, we need to involve Filipino GBM in shaping health decision-making processes.

We also need to democratize the expertise of HIV and mental health in the Philippines by involving people with lived experiences with HIV and/or mental health conditions in their health decision-making and, at the same time, use their experiential expertise to help improve service delivery, make decisions at a policy level, and increase representation and involvement through co-designing services, programs, and interventions. Past evidence showed that community-based HIV services in the Philippines through The LoveYourself—a community-based and volunteer-led organization addressing the HIV epidemic in the Philippines—showed better HIV viral suppression and initiation of anti-retroviral therapy compared to the Philippines’ national outcomes [46].

Indeed, there has been an increasing momentum in the lived experience research [e.g., 47]. However, we need to be mindful of lived experience representations [see 48] in HIV and mental health spaces since institutions still fail to include peoples’ lived experiences in service delivery, co-design, and decision-making, which may lead to tokenistic practices [49]. Nonetheless, we need to shift the power and expertise towards those who are living with HIV and mental health conditions.

Past literature showed that Filipino GBM, as well as other men who have sex with men, have faced marginalization and inequities, particularly when seeking access to treatment and health services. Hostile treatment facilities have made it difficult for them to access the care they need [50], and healthcare workers have limited knowledge about the health challenges among this population [51]. Additionally, societal views influenced by Roman Catholic teachings have contributed to the perception of these men as morally wrong and sinful [52]. As a result, many struggle with their identities, reconciling their sexuality with societal and religious expectations [53]. Despite the struggles of navigating their identities from the conservative values of Roman Catholicism, past evidence showed that they managed to incorporate their sexual identity into an LGBTQIA+ inclusive church and communities [54]. Nevertheless, it is long overdue to let these men be well-represented in their health—including their decision-making and see them as leaders who would inform and be involved in policy and decision-making about their health. Doing so will enable us to create meaningful and inclusive healthcare services and interventions. For instance, when healthcare workers and policymakers involve people by including their lived experiences in shaping health service provisions, healthcare workers can learn from them and create interventions suitable for this population. At the same time, policymakers can develop policies guided by the experiences of this population and involve them in decision-making initiatives.

#### 2. Emphasize social, structural, and political elements of health conditions

We must go beyond the biomedical approaches to HIV and mental health conditions and embrace the plurality of perspectives, including the social constructions of health conditions. As researchers and healthcare workers, we must situate how health conditions co-exist with other health conditions and other social and structural determinants. We need to deviate from looking at health conditions from a single-axis view to a pluralistic approach; that is, health conditions co-occur with other health conditions. We also invite other stakeholders in this area to reflect on their practices and consider the social, structural, and political elements of HIV and mental health.

In the context of the Filipino MSM community, HIV often co-exists with problems in mental health [e.g., 55] and other health conditions [e.g., 56]. Embracing intersectionality to complement syndemics in understanding the comorbidity of two or more health conditions encourages us to examine how political, religious, and structural elements have shaped the HIV and mental health outcomes among the Filipino MSM community. This approach prompts critical questions, such as: How do economic inequalities and social class, traditional Filipino culture, and collectivistic society contribute to the ongoing HIV and mental health problems? How do cisheteronormativity, sexism, and conservative sexual attitudes contribute to the inaccessibility of healthcare service provisions on HIV and mental health? What is the community’s role in promoting or addressing the ongoing marginalization among Filipino MSM? How do the intersections of political polarization and unclear demarcations of the Church and government interact with that aim to protect the multiply marginalized populations? For us to understand and create solutions through interventions toward the disproportionately multiply marginalized populations in the Philippines, including the MSM community, we need collaboration that transcends our distinct disciplines and embraces multidisciplinary partnerships with other experts outside our discipline.

One significant barrier to applying syndemics in this population is the limited resources for conducting research and securing funding. To the best of our knowledge, the application of syndemics among Filipino MSM and within the Philippines is still in its early stages, with few researchers engaged in this work. We hope this essay catalyzes further discourse and inspires more scholars to engage in syndemics research.

Healthcare models in many Asian societies often operate in a paternalistic framework, where medical health professionals are perceived as the sole authorities in the healthcare provision [57]. This dynamic reinforces power imbalances and excludes the voices of people with lived experiences of health conditions [58], including HIV and mental health issues. To address this limitation, we need to shift these dynamics by involving individuals with lived experiences in healthcare models, ensuring that their insights inform and enhance HIV and mental healthcare practices.

#### 3. Address stigma

To craft interventions aimed at addressing HIV and mental health issues among Filipino MSM, we must tackle stigma and create culturally informed stigma reduction strategies. To develop these strategies, we must first understand the complexity of stigma experiences among Filipino MSM. Once identified, we need to create opportunities for them to be empowered by including them at *higher* levels of the HIV and mental health systems. This approach seeks to shift the power balance from healthcare providers to the community, recognizing their experiential expertise in HIV and mental health.

Contemporary social scientists described stigma as a process where a person deemed different is given a mark that they are undesirable or discredited, leading to exclusion, discrimination, and abuse [59]. Scholars view stigma as dynamic [59] and multifaceted [60]. However, scholars [61] pointed out two challenges of conceptualizing stigma. First, scholars often misunderstand the experiences of people with stigmatized identities because they are cultural outsiders (i.e., not part of the stigmatized group) and are often naive about their lived experiences [62]. Second, scholarly work on stigma deliberately narrows down its focus on individual perception and the outcomes of such perceptions [63].

Evidence suggests stigma remains one of the barriers to accessing treatment and care for HIV [e.g., 50]. However, past works on stigma failed to acknowledge the multiplicity of stigma, such as how it is a dynamic process that changes over time [64]. Understanding the complexity of stigma requires the use of critical lenses. Expanding on the earlier discussions, we emphasize that intersectionality might provide a framework to understand how various structural elements of oppression interlock and influence stigma among Filipino GBM [37,38]. However, we must be cautious in using intersectionality in research as some scholarly works that claim to use intersectionality only identify the multiple stigmatized identities of multiply marginalized populations and fail to recognize how interlocking forces of oppression shaped this stigma, which could lead to the categorization of social identities [41]. We underscore the importance of research that recognizes the significance of intersectionality in understanding stigma and its role in HIV and mental health outcomes. However, engaging in intersectionality research necessitates accountability in identifying how social, political, and structural forces shape marginalization. This is fundamental to creating inclusive programs and interventions for Filipino MSM.

#### 4. Commit to a therapeutic alliance

Healthcare workers in HIV and mental health must commit to achieving a therapeutic alliance with their clients, including Filipino MSM, and place paramount emphasis on the cultural nuances of the community of Filipino MSM. Disconnection happens between healthcare workers and their gay and or bisexual men clients because healthcare workers are often unaware of the struggles of their clients [51]. This lack of understanding can prevent the formation of a therapeutic alliance, as trust is not established [65]. For instance, people living with HIV in the Philippines had poor treatment adherence because HIV healthcare facilities were unsupportive of their privacy in accessing such health services [50]. Additionally, evidence showed that the healthcare workers’ lack of awareness of the struggles of Filipino GBM led to detrimental attitudes toward them [51]. However, we need to be critical regarding the therapeutic alliance. It is not necessarily the case that establishing a therapeutic alliance automatically leads to better health outcomes.

## Conclusion

This essay aims to illustrate how the use of critical lenses of syndemics and intersectionality would enable stakeholders to contextualize and address the ongoing HIV and mental health problems in the Philippines. Filipino MSM have been disproportionately burdened by HIV and mental health issues, which are fueled by their multiple stigmatized identities and systemic oppression and marginalization. By embedding syndemics and intersectionality theories in research and praxis, stakeholders can capture the nuanced experiences of living with these health conditions and stigmatized identities. This approach would also help identify how socio-political and structural elements have shaped their health conditions and experiences. Ultimately, we hope to encourage a critical approach to health that creates holistic, inclusive, and culturally informed programs and interventions for Filipino MSM.
